# Which is the best diet to reduce cardiometabolic risk: dietary counseling or home-delivered diet?

**DOI:** 10.29219/fnr.v67.9855

**Published:** 2023-12-27

**Authors:** Feray Çağiran Yilmaz, Aysun Atilgan, Günay Saka

**Affiliations:** 1 Department of Nutrition and Dietetics, Faculty of Health Sciences, Dicle University, Diyarbakır, Turkey; 2 Department of Public Health, Faculty of Medicine, Dicle University, Diyarbakır, Turkey

**Keywords:** home-delivered diet, dietary compliance, dietary counseling, obesity, cardiometabolic risk

## Abstract

**Background:**

Non-compliance with medical nutrition therapy can lead to an increase in cardiometabolic risk factors, resulting in an increase in the frequency of morbidity and mortality.

**Objective:**

This study aims to compare the effectiveness of two different medical nutrition therapies designed to reduce cardiometabolic risk factors.

**Design:**

The study was conducted on voluntary overweight and obese women who sought services at a specialized Nutrition and Dietary Counseling Clinic. The clinic offered dietary counseling (*n* = 47) and home-delivered diet (*n* = 53) services, and the participants themselves decided which method they preferred. Both groups were followed for a period of 4 weeks. The general information, physical activity (PA) status, biochemical findings, blood pressure, anthropometric measurements, and bioelectrical impedance analysis (BIA) of the voluntary participants were evaluated. Taking into consideration the participants’ dietary habits and health status, an appropriate dietary plan (55–60% carbohydrates, 15–20 protein%, 25–30% fat) was prepared.

**Results:**

The anthropometric measurements, blood pressure, and biochemical parameters of overweight and obese individuals who received dietary counseling and home-delivered diet services were evaluated before and after the diet. In both groups, cardiometabolic risk factors were found to decrease. It was determined that those who received the home-delivered diet service had a greater reduction in body weight, Body Mass Index values, body fat percentages, and waist circumferences compared to those who received dietary counseling (*P* < 0.001). Similarly, fasting blood glucose, triglyceride, and blood pressure values were found to decrease more significantly in individuals receiving the home-delivered diet service (*P* < 0.001). Additionally, both groups showed an increase in High-Density Lipoprotein Cholesterol (HDL-C) levels, with a greater increase observed in those receiving the home-delivered diet service (*P* < 0.001).

**Conclusion:**

According to the findings of this study, participants who received the home-delivered diet service, which is particularly beneficial for individuals who struggle with healthy meal preparation and portion control, exhibited better adherence to medical nutrition therapy and experienced a greater reduction in cardiometabolic risk factors compared to those who received dietary counseling.

## Popular scientific summary

- Even though there are studies showing that medical nutrition therapy is effective in reducing cardiometabolic risk factors, the number of studies examining different dietary approaches are limited.
- This study found that both dietary counseling and home-delivered diet services reduced cardiometabolic risk factors.
- However, it was determined that individuals adapted better to home-delivered diet service and there was a greater decrease in cardiometabolic risk factors compared to diet counseling.

## Key points

In this study, the effects of two different diets on cardiometabolic risk were examined. It has been determined that home-delivered diet is a better choice than dietary compliance in reducing cardiometabolic risk.

Chronic diseases associated with obesity contribute to increased morbidity and mortality risks worldwide (1,2,3). Obesity, along with cardiometabolic risk factors such as hyperglycemia, dyslipidemia, and hypertension, can be modified through diet and other lifestyle interventions (4,5,6). Therefore, many healthcare organizations recommend medical nutrition therapy provided by dietitians for dietary and lifestyle changes (6, 7). Medical nutrition therapy is the initial treatment option that enables individuals to lose weight, reduce fat mass, and improve blood parameters in a healthy manner. Numerous studies demonstrated the effectiveness of medical nutrition therapy (8,9,10,11,12).

Genetic variations, lack of knowledge and skills to modify the diet, difficulty in adhering to the diet, and inadequate time for preparing healthy meals are among the reasons that may hinder the desired response to medical nutrition therapy. While dietary counseling under the guidance of a professional Nutrition Specialist can yield positive outcomes in obesity treatment, maintaining the diet and preserving the achieved results can be challenging for individuals (13,14,15).

In the dietary counseling method, a calorie is determined according to the age, gender, and body mass index (BMI) value of the people who apply to the dietitian, and a diet list appropriate to the living conditions of the individuals is prepared by the dietitian. Compliance with the diet lists prepared specifically for individuals is monitored by the dietitian (16). In the home-delivered diet, the amount of energy and macro nutrients that individuals should consume daily is calculated by the dietitian. Meals prepared according to these values are delivered fresh to people’s addresses every day (17).

Traditional strategies used in dietary counseling involve behavioral approaches such as goal setting, weekly check-ins, tracking charts, and the preparation of diet lists by dietitians to improve dietary adherence. Adhering to such intensive programs can be quite challenging for individuals. Researchers demonstrated that individuals struggle to make appropriate food choices when eating out or at home, regardless of their nutrition education. Ready-to-eat, diet-compliant, balanced, diverse, and calorie-controlled diet meals delivered to the specified address can provide a new perspective in preventing obesity and lead to effective results. In this context, the implementation of home-delivered diet meals aims to prevent obesity and obesity-related chronic diseases (17,18,19,20,21,22).

The importance of medical nutrition therapy of chronic diseases cannot be denied. Dietary counseling is a method that has been applied by dietitians and nutritionists for many years and provides successful results. However, the acceleration of life has revealed the necessity of an easier and more practical method other than the dietary counseling method. In the early years, the home-delivered diet method, which targeted balanced and varied nutrition for the elderly people who had difficulty accessing food, is also applied to individuals with chronic nutrition-related diseases such as heart diseases, diabetes, and obesity (20, 23, 24). This study aims to compare the effectiveness of two different medical nutrition therapies, dietary counseling and home-delivered diet, designed to reduce cardiometabolic risk factors in overweight and obese women.

## Method and analyses

### Study design and data collection

Individuals who applied to a well-known specialized diet clinic where ‘dietary counseling’ and ‘home-delivered diet’ methods are provided were informed about both weight loss methods and expressed their willingness to participate in the study as volunteers were included in the study. The participants were overweight and obese women who desired to lose weight. Pregnant and lactating women, alcohol users, individuals with chronic health conditions taking medication, those using weight loss drugs or steroids, and individuals with disabilities were excluded from the study.

The participants were informed about the dietary counseling and home-delivered diet service programs, and they were given the freedom to choose which program they wanted to participate in. Prior to the study, the participants were asked to keep a 3-day food intake record, and they were instructed to come to the clinic after an 8-h fasting period, along with their 3-day food intake records. Blood samples and blood pressure measurements of the participants were collected by an experienced nurse, while their sociodemographic characteristics, dietary history, anthropometric measurements, and Bioelectrical Impedance Analysis (BIA) data were recorded by a dietitian.

The study obtained ethical approval from the [removed for blind peer review]. The participants were provided with detailed information about the research and were then asked to sign an Informed Consent Form. The study was conducted following the guidelines outlined in the Helsinki Declaration.

### Dietary counseling

In the dietary counseling service, the participant’s necessary measurements were taken, and their daily energy requirement was determined. The daily energy requirement was calculated using the formula, Basal Metabolic Rate (BMR) × Physical Activity (PA). The World Health Organization (WHO) equation was used to determine the BMR (25). The daily energy requirement was then reduced by 500 kilocalories. Before starting the diet and after the diet, blood tests were requested. The diet consisted of three main meals and two snacks, totaling five meals. The individual was provided with a diet plan based on these guidelines. The individual prepared their meals according to the diet plan. The daily energy intake was composed of 55–60% carbohydrates, 12–15% proteins, 25–30% fats and total dietary fiber intake was 25 to 30 g.

### Home-delivered diet service

In the home-delivered diet service, the patient’s necessary measurements and examinations were conducted, and their daily energy requirement was determined. The daily energy requirement was calculated using the formula, BMR × PA. The WHO equation was used to determine the BMR (25). The daily energy requirement was then reduced by 500 kilocalories. Before starting the diet and after the diet, blood tests were requested. The diet consisted of three main meals and two snacks, totaling five meals. In line with this, the specific diet prepared for the individual was prepared and delivered to the individual’s address under suitable conditions. The daily energy intake was composed of 55–60% carbohydrates, 12–15% proteins, 25–30% fats, and total dietary fiber intake was 25–30 g. In the home delivery diet service, meals prepared by experienced chefs under the supervision of a dietitian are delivered to the participants’ homes ready-made.

### Anthropometric measurements

The anthropometric measurements of the individuals were taken twice, once before starting the diet and again 1 month after the diet. The researcher measured the individual’s body weight (kg), height (cm), waist circumference (cm), hip circumference (cm), upper mid-arm circumference (cm), and neck circumference (cm). Body weight measurement was taken in the morning on an empty stomach, with light clothing and without shoes. Height measurement was taken with a fixed tape measure, with the heels touching the wall, shoulder and back against the wall, and the feet together. Waist circumference was measured by finding the midpoint between the lowest rib and the iliac crest, and neck circumference was measured from the lower border of the laryngeal prominence (Adam’s apple). Hip circumference was measured at the highest point of the hip after the individual was turned sideways (26). Upper mid-arm circumference was measured with the individual standing upright, the elbow flexed at a 90° angle, and the midpoint between the acromion process and the elbow marked and measured with a tape measure (27).

BMI was calculated by dividing the individual’s body weight (kg) by the square of their height (in meters) (BMI = kg/m^2^). Individuals with a BMI between 18.5 and 24.9 kg/m^2^ were classified as normal, those with a BMI between 25.0 and 29.9 kg/m^2^ were classified as overweight, and those with a BMI of 30 kg/m^2^ or above were classified as obese (25).

In addition, at the initial consultation and after 1 month, measurements were taken using the InBody-270 BIA device to record body weight, lean body mass, body fat weight, and body fat percentage. Before the measurements, the device electrodes were cleaned with pure alcohol. The purpose and content of the measurements were explained to the individuals before the measurement.

Prior to the measurement, individuals were instructed to fast for 8 h and avoid excessive consumption of diuretic fluids such as water, tea, and coffee for at least 4 h before the measurement. They were also advised to refrain from intense exercise. Individuals were instructed to remove any metal items, such as rings or bracelets, that came into contact with their bodies before the measurement. Care was taken to ensure that individuals maintained the appropriate position on the measurement device. Measurements were not taken during the menstrual period for women, as hormonal changes during menstruation can affect body weight (28).

### Biochemical measurements

The participants in the study were asked to provide fasting blood samples at their respective Family Health Centers. All blood samples were collected by experienced nurses in the morning after an 8–12 h fasting period. After collection, the tubes were gently shaken and centrifuged for 10–15 min (at 3,200 rpm) and then stored at –80°C. The participants were instructed to provide blood samples twice: at the beginning of the study and at the end of the first month. Biochemical tests were performed using the Cobas 6000 E 501 analyzer from Roche Diagnostics GmbH, Mannheim, Germany. The participants’ HOMA-IR (Homeostasis Model Assessment of Insulin Resistance) values were calculated using the formula: fasting plasma glucose (mmol/L) multiplied by fasting plasma insulin levels (μU/mL), divided by 22.5 (19).

### Statistical analysis of the data

The data were statistically analyzed using the Statistical Package for the Social Sciences 25 (SPSS-25) program. For the comparison of categorical data and the evaluation of changes within groups, the Pearson chi-square test was used when the number of cells with expected frequencies less than 5 did not exceed 20% of the total cells, and the Fisher’s exact test was used when it exceeded 20%. The normal distribution of quantitative data was assessed using the Kolmogorov–Smirnov and Shapiro–Wilk tests. When two groups exhibited a normal distribution, the t-test was used to compare their means, and descriptive statistics such as mean (X), standard deviation (SD), minimum, and maximum values were reported. A confidence interval of 95% was used for all statistical tests and interpretations.

## Results

The individual characteristics of the participants according to the type of diet are presented in Table 1. All participants in the study were female, and the majority were married. It was found that the average age of individuals receiving dietary counseling was higher compared to those receiving home-delivered diet services (*P* < 0.05). There were no statistically significant differences between the groups in terms of education level, occupation, income level, previous dieting experience, regular PA status and frequency, and daily sleep duration (*P* > 0.05).

**Table 1:** Individual characteristics of participants according to diet type

General information		Dietary compliance	Home-delivered diet	*P*
Age (X ± SD)	Years	36.3 ± 9.8	31.2 ± 7.5	0.004*
Gender, *N* (%)	Female	100 (100.0)	100 (100.0)	-
Marital status, *N* (%)	Married	32 (68.1)	29 (54.7)	0.219**
	Single	15 (31.9)	24 (45.3)	
Educational level, *N* (%)	High school graduate	7 (14.9)	4 (7.5)	0.418**
	University student	4 (8.5)	7 (13.2)	
	University graduate	36 (76.6)	42 (79.2)	
Occupation, *N* (%)	Housewife	12 (25.5)	3 (5.7)	0.061**
	Civil servant	30 (63.8)	38 (71.7)	
	Self-employed	1 (2.1)	5 (9.4)	
	Student	4 (8.5)	7 (13.2)	
Income level, *N* (%)	Moderate	10 (21.3)	6 (11.3)	0.284**
	High	37 (78.7)	47 (88.7)	
Previous dieting experience, *N* (%)	Yes	23 (48.9)	29 (54.7)	0.564**
	No	24 (51.1)	24 (45.3)	
Regular physical activity, *N* (%)	Yes	8 (17.0)	3 (5.7)	0.070**
	No	39 (83.0)	50 (94.3)	
Frequency of regular physical activity, (X ± SD) (Min–Max)	Minutes	39.1 ± 9.9 (30.2–60.5)	46.9 ± 11.6 (40.2–60.3)	0.297*
Average daily sleep duration, (X ± SD) (Min–Max)	Hours/day	7.1 ± 0.7 (6.0–9.0)	6.9 ± 0.7 (5.0–9.0)	0.074*

*p* < 0.005.

* Independent *t*-test.

** Pearson chi-square test.

Table 2 provides the pre-diet and post-diet anthropometric measurements of the participants according to their diet type. Both groups experienced a decrease in all anthropometric measurements after the diet. It was observed that the average difference between pre-diet and post-diet measurements was significantly higher in the group receiving home-delivered diet services compared to the group receiving dietary counseling (*P* < 0.001).

**Table 2:** Pre-diet and post-diet anthropometric measurements according to diet type

Anthropometric measurements	Dietary compliance			Home-delivered diet			*P* *
	Pre-diet	After 4 weeks of diet	Mean difference	Post-diet	After 4 weeks of diet	Mean difference	
Body weight (kg) (X ± SD)	86.1 ± 14.4	81.1 ± 13.9	5.0 ± 0.5	85.2 ± 18.9	79.9 ± 18.1	5.3 ± 0.8	<0.001
Height (cm)(X ± SD)	162.5 ± 7.1						-
BMI (kg/m^2^) (X ± SD)	32.6 ± 4.7	30.5 ± 4.7	2.1 ± 0.0	31.4 ± 5.7	29.2 ± 5.4	2.2 ± 0.3	<0.001
Waist circumference (cm) (X ± SD)	97.0 ± 15.9	91.8 ± 15.8	5.2 ± 0.1	99.3 ± 12.9	93.2 ± 12.5	6.1 ± 0.4	<0.001
Hip circumference (cm) (X ± SD)	114.9 ± 12.6	109.5 ± 11.5	5.4 ± 1.1	114.7 ± 8.5	109.2 ± 8.7	5.5 ± 0.2	<0.001
Upper mid-arm circumference (cm) (X ± SD)	33.4 ± 4.6	31.1 ± 4.6	2.3 ± 0.0	34.0 ± 3.8	31.6 ± 3.8	2.4 ± 0.0	<0.001
Neck circumference (cm) (X ± SD)	35.8 ± 3.8	33.7 ± 3.7	2.1 ± 0.1	36.7 ± 3.4	34.3 ± 3.5	2.4 ± 0.1	<0.001
Lean body mass (kg) (X ± SD)	27.2 ± 5.4	26.4 ± 5.2	0.8 ± 0.2	27.3 ± 5.6	26.3 ± 5.4	1.0 ± 0.2	<0.001
Fat mass (kg) (X ± SD)	37.2 ± 8.5	33.5 ± 8.8	3.7 ± 0.3	35.9 ± 12.4	31.9 ± 11.6	4.0 ± 0.8	<0.001
Body fat percentage (%) (X ± SD)	42.9 ± 5.2	40.9 ± 5.8	2.0 ± 0.6	41.6 ± 6.8	38.9 ± 6.7	2.7 ± 0.1	<0.001

* Independent *t*-test (difference between mean differences).

Table 3 presents information regarding pre-diet and post-diet biochemical measurements and blood pressures according to the diet type. In both groups, post-diet measurements showed a decrease in fasting blood glucose, Blood Urea Nitrogen (BUN), Aspartate Aminotransferase (AST), Alanine Aminotransferase (ALT), cholesterol, Triglyceride (TG), Low Density Lipoprotein Cholesterol (LDL), Thyroid-Stimulating Hormone (TSH), Hemoglobin A1C (HbA1c), C-Reactive Protein (CRP), creatinine, insulin, Homeostatic Model Assessment of Insulin Resistance (HOMA-IR), diastolic blood pressure (DBP), and systolic blood pressure (SBP) values, while High-Density Lipoprotein Cholesterol (HDL), ferritin, vitamin B12, and vitamin D levels were found to increase. It was determined that the reductions and elevations in the group receiving home-delivered diet services were statistically higher compared to the dietary counseling group (*P* < 0.001).

**Table 3:** Pre-diet and post-diet biochemical measurements and blood pressures according to diet type

Biochemical measurements	Dietary compliance			Home-delivered diet			*P* *
	Pre-diet	After 4 weeks of diet	Mean difference	Post-diet	After 4 weeks of diet	Mean difference	
Fasting Blood Glucose (mg/dL) (X ± SD)	102.9 ± 23.2	96.5 ± 21.8	6.4 ± 1.4	98.9 ± 13.7	92.0 ± 10.6	6.9 ± 3.1	<0.001
BUN (mg/dL) (X ± SD)	19.2 ± 7.0	17.5 ± 6.7	1.7 ± 0.3	18.5 ± 7.5	16.5 ± 6.5	2.0 ± 1.0	<0.001
AST (IU/L) (X ± SD)	21.5 ± 8.4	19.6 ± 7.9	1.9 ± 0.5	25.6 ± 9.5	23.3 ± 8.3	2.3 ± 1.2	<0.001
ALT (U/L)(X ± SD)	20.8 ± 9.5	18.8 ± 8.9	2.0 ± 0.6	29.9 ± 22.9	27.3 ± 21.2	2.6 ± 1.7	<0.001
Cholesterol (mg/dL) (X ± SD)	182.7 ± 37.9	162.9 ± 30.8	19.8 ± 7.1	197.9 ± 47.7	175.9 ± 41.5	22.0 ± 6.2	<0.001
TG (mg/dL) (X ± SD)	142.3 ± 73.9	127.3 ± 63.2	15.0 ± 10.7	146.0 ± 69.8	123.2 ± 54.6	22.8 ± 15.2	<0.001
LDL (U/L) (X ± SD)	123.3 ± 46.2	111.1 ± 39.9	12.2 ± 6.3	133.9 ± 47.1	120.9 ± 41.2	13.0 ± 5.9	<0.001
HDL (mg/dL)	45.6 ± 15.2	47.9 ± 14.1	2.3 ± 1.1	42.9 ± 10.3	46.1 ± 10.3	3.2 ± 0.0	<0.001
Ferritin (mL/ng) (X ± SD)	57.5 ± 34.7	63.8 ± 35.6	6.3 ± 0.9	61.9 ± 36.9	70.0 ± 40.6	8.1 ± 3.7	<0.001
B12 (pg/mL) (X ± SD)	257.3 ± 124.9	279.6 ± 132.9	22.3 ± 8.0	305.3 ± 137.4	334.5 ± 156.1	29.2 ± 18.7	<0.001
TSH (IU/L)(X ± SD)	1.6 ± 1.5	1.4 ± 1.4	0.2 ± 0.1	1.4 ± 1.0	1.1 ± 0.8	0.3 ± 0.2	<0.001
HbA1c (mmol/mol)(X ± SD)	5.4 ± 0.9	5.1 ± 0.8	0.3 ± 0.1	5.5 ± 0.8	5.1 ± 0.7	0.4 ± 0.1	<0.001
CRP (mg/L)(X ± SD)	1.6 ± 1.5	1.3 ± 1.1	0.3 ± 0.4	2.7 ± 3.9	1.9 ± 2.7	0.8 ± 1.2	<0.001
Vitamin D (ng/mL)(X ± SD)	32.7 ± 16.9	36.2 ± 12.9	3.5 ± 4.0	30.8 ± 8.4	35.7 ± 8.8	5.1 ± 0.4	<0.001
Creatinine (mg/dL)(X ± SD)	0.8 ± 0.3	0.8 ± 0.2	0.0 ± 0.1	0.9 ± 0.6	0.8 ± 0.2	0.1 ± 0.4	<0.001
Insulin (%)(X ± SD)	11.3 ± 5.1	10.1 ± 4.5	1.2 ± 0.6	10.5 ± 3.1	9.2 ± 2.1	1.3 ± 1.0	<0.001
HOMA-IR (mmol/L)(X ± SD)	2.9 ± 1.5	2.4 ± 1.3	0.5 ± 0.2	2.8 ± 0.9	2.1 ± 0.6	0.7 ± 0.3	<0.001
DBP (mmHg)(X ± SD)	82.6 ± 1.2	81.9 ± 0.9	0.7 ± 0.3	84.3 ± 1.7	82.5 ± 1.3	1.8 ± 0.4	<0.001
SBP (mmHg)(X ± SD)	118.7 ± 1.2	117.9 ± 0.9	0.8 ± 0.3	121.4 ± 1.6	119.8 ± 1.2	1.6 ± 0.4	<0.001

* Independent *t*-test (difference between mean differences).

**Fig. 1:**
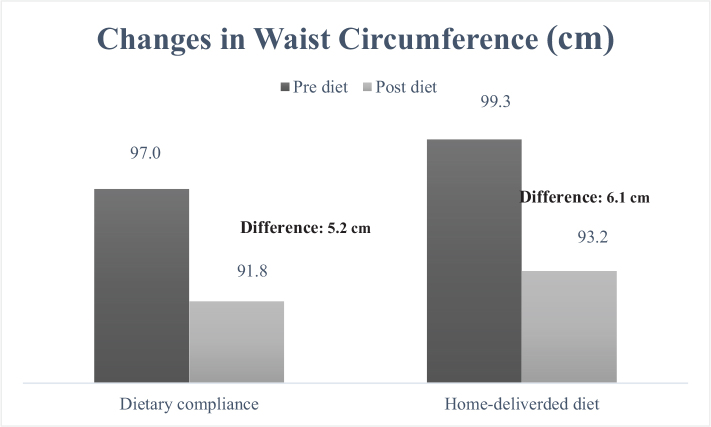
Changes in waist circumference of individuals according to diet types.

**Fig. 2:**
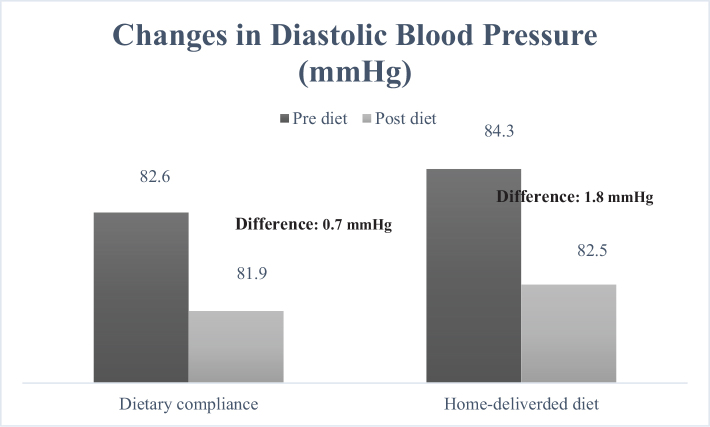
Changes in diastolic blood pressure of individuals according to diet types.

**Fig. 3:**
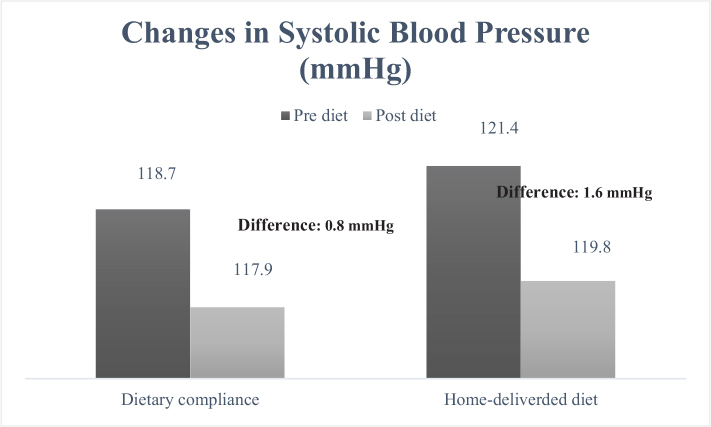
Changes in systolic blood pressure of individuals according to diet types.

**Fig. 4:**
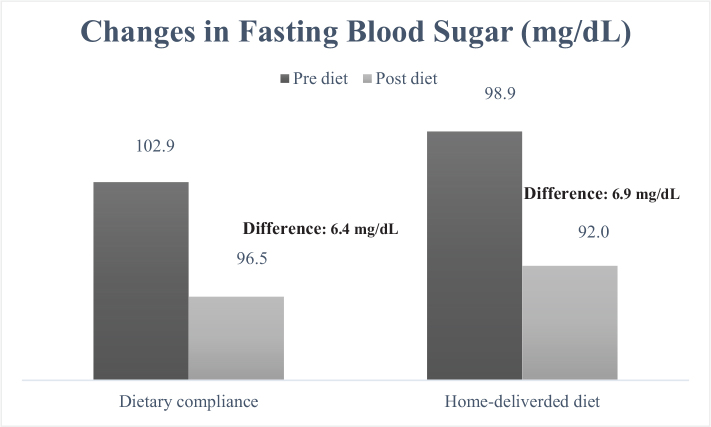
Changes in fasting blood sugar of individuals according to diet types.

**Fig. 5:**
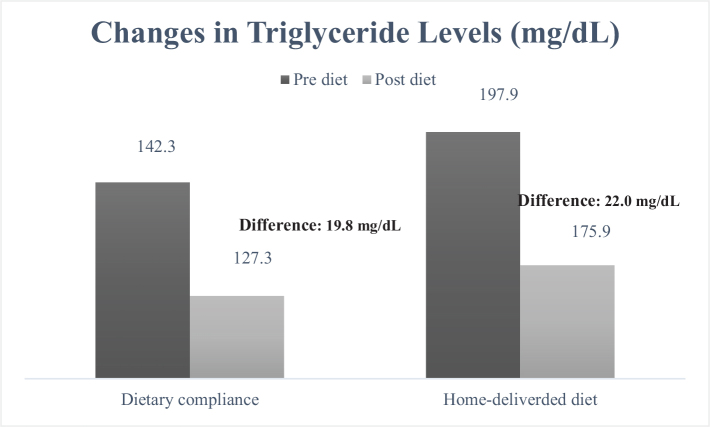
Changes in triglyceride levels of individuals according to diet types.

**Fig. 6:**
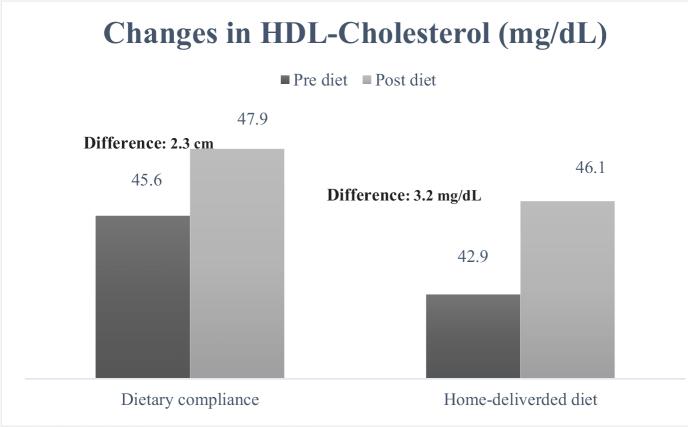
Changes in HDL-cholesterol levels of individuals according to diet types.

When examining the cardiometabolic risk factors individually, the measurement of waist circumference (Fig. 1) decreased from 99.3 to 93.2 cm in the group receiving home-delivered diet services (mean difference: 6.1 cm), while in the dietary counseling group, this value decreased from 97.0 to 91.8 cm (mean difference: 5.2 cm). It was determined that the decrease was greater in the group receiving home-delivered diet services (*P* < 0.001). When looking at the differences in blood pressure between the groups (Figs 2 and 3), both DBP and SBP showed a greater decrease in the group receiving home-delivered diet services (*P* < 0.001). Figure 4 presents the differences in fasting blood sugar levels of individuals according to diet types. In the group receiving home-delivered diet services, fasting blood sugar decreased by an average of 6.9 mg/dL, while in the dietary counseling group, it decreased by 6.4 mg/dL (*P* < 0.001). Both groups showed a decrease in triglyceride levels after the diet (Fig. 5), but the decrease was statistically greater in the group receiving home-delivered diet services (*P* < 0.001). As seen in Fig. 6, both groups experienced an increase in HDL cholesterol levels after the diet, with a greater increase observed in the group receiving home-delivered diet services (*P* < 0.001).

## Discussion

Obesity continues to be a significant risk factor for nutrition-related chronic diseases such as cardiovascular disease, hypertension, coronary heart disease, type 2 diabetes, and cancer, making it a serious public health issue. The prevalence of obesity approximately tripled between 1975 and 2016, reaching pandemic proportions in recent years. The WHO reports that approximately 13% of the world’s population, or over 650 million adults, are affected by obesity and obesity-related chronic diseases. It is estimated that the number of individuals affected by obesity will exceed 2 billion by 2035 (29,30,31,32).

Non-adherence to medical nutrition therapy, which is the primary approach for preventing nutrition-related chronic diseases, increases the morbidity and mortality risk. There are various reasons for non-adherence to medical nutrition therapy, including the difficulty of diets, limited dietary variety, insufficient time and knowledge for meal preparation, and the restrictive nature of many diets. There is a need for simpler methods to improve adherence to diets. In recent years, the concept of the home-delivered diet has gained attention as a potential solution to this problem, leading to an increase in related studies (33,34,35,36,37).

As previously reported in studies, reducing dietary fat, saturated fat, and cholesterol can lower individuals’ cardiovascular disease risk by decreasing their blood lipid levels. Today, one of the most challenging aspects for healthcare professionals is motivating patients to make necessary dietary changes and ensuring their continued adherence to these changes. The planned home-delivered diet service in this study aims to overcome these challenges and develop an approach that can serve as an example for large healthcare organizations. With the home-delivered diet service, individuals can reduce their obesity and obesity-related chronic disease risk by consuming the delivered meals without having to worry about confusing and knowledge-demanding tasks such as shopping, meal preparation, using appropriate cooking methods, and determining the calorie content of meals.

In both diet approaches planned in this study for reducing cardiometabolic risk factors, approximately 55–60% of daily energy intake was provided from carbohydrates, 12–15% from proteins, and 25–30% from fats. The energy intake was reduced by 500 kcal in both groups. Subsequently, the patients’ anthropometric measurements, biochemical findings, and cardiometabolic risk factors were examined.

The process of dietary counseling can be disruptive and time-consuming, which can negatively affect an individual’s determination to maintain changes. Providing meals that meet the individual’s nutritional needs through a home-delivered diet service can reduce these difficulties. Adherence to a ready-made meal plan improves overall health, daily activities, work performance, and perceptions of nutritional health while reducing concerns and improving mental health due to decreased anxiety. A study revealed that individuals who received a home-delivered diet service had higher dietary fat and saturated fat intake compared to those who received dietary counseling. It was noted that individuals receiving the home-delivered diet service showed better adherence to their diets, resulting in greater reductions in blood parameters. In our study, individuals who received the home-delivered diet service showed greater reductions in anthropometric measurements, blood pressure, and blood lipids. The easier adherence to the diet and the more controlled nature of the home-delivered diet service could be considered as the reasons for this outcome (38).

In previous studies, it was reported that high abdominal fat accumulation is a significant risk factor for cardiometabolic diseases (39), and it was demonstrated that waist circumference is inversely associated with the risk of type 2 diabetes mellitus (40). A meta-analysis study indicated that abdominal obesity is a better indicator than BMI for identifying cardiovascular diseases (41). In a study examining cardiovascular disease risk, individuals receiving home-delivered diet services showed a reduction of 5.08 ± 0.05 cm in waist circumference and 3.30 ± 0.05 cm in hip circumference after an 8-week follow-up (15). Our study also demonstrated a decrease in waist circumference, hip circumference, lean body mass, fat mass, and percentage in both the group receiving dietary counseling and the group receiving home-delivered diet services, with a greater reduction observed in the latter group (*P* < 0.001).

In a randomized controlled trial, hypertensive, dyslipidemic, or diabetic patients receiving outpatient treatment were provided with a home-delivered diet service following the recommendations of the American Diabetes Association. After a 10-week period, it was found that these individuals experienced greater improvements in lipid profile, blood glucose levels, and body weight compared to the group receiving dietary counseling (42). Another study conducted with individuals with cardiovascular disease showed that those receiving home-delivered diet services had greater reductions in body weight, waist circumference, and hip circumference, as well as improvements in lipid profile compared to those receiving dietary counseling (15). A randomized trial focusing on low-fat and low-glycemic index home-delivered diet services for obese individuals demonstrated significant improvements in cardiometabolic risk factors, particularly notable reductions in adiposity, lipid measurements, and blood pressure (43). Similarly, in our study, significant improvements in cardiometabolic risk factors were observed.

Longer-term studies would be beneficial to determine if patients with coronary artery disease can adhere to such a program for several months and continue to reduce their cardiovascular risks. Metz et al. conducted a study in which overweight and obese patients were provided with a long-term home-delivered diet service, and it was found that at the end of the program, they lost an average of 5.8 kg, experienced reductions in cholesterol and LDL levels, and an increase in HDL levels (44). Although our study had a shorter duration, the results obtained after just 4 weeks were quite valuable. Conducting a longer-term study would be important to assess the effectiveness of the intervention. Additionally, our study was conducted at a single center and included only women. It is recommended to conduct multicenter studies involving both genders to evaluate the results within a greater scope.

## Conclusions

Obesity is a rapidly increasing global public health problem with a high prevalence worldwide. It is a leading cause of preventable deaths and contributes to the development of chronic diseases. A significant majority of obese individuals struggle with non-compliance to medical nutrition therapy, citing reasons such as difficulty in finding the necessary food items for healthy meal preparation, lack of access to healthy food during meal times, inability to portion control, insufficient knowledge about healthy food preparation, and lack of time to prepare personalized meals. This study demonstrates that home-delivered diet services are an important approach for improving health and promoting behavior change, and they are more successful in reducing cardiometabolic risk factors compared to dietary counseling.

## Transparency declaration

The lead author affirms that this manuscript is an honest, accurate, and transparent account of the study being reported. The reporting of this work is compliant with PRISMA guidelines. The lead author affirms that no important aspects of the study have been omitted and that any discrepancies from the study as planned have been explained. The review was not registered.
